# Synthesis route and three different core-shell impacts on magnetic characterization of gadolinium oxide-based nanoparticles as new contrast agents for molecular magnetic resonance imaging

**DOI:** 10.1186/1556-276X-7-549

**Published:** 2012-10-03

**Authors:** Gholamreza Azizian, Nader Riyahi-Alam, Soheila Haghgoo, Hamid Reza Moghimi, Reza Zohdiaghdam, Behrooz Rafiei, Ensieh Gorji

**Affiliations:** 1 Medical Physics & Biomedical Engineering Department, School of Medicine, Tehran University of Medical Sciences (TUMS), Keshavaz blvd, 16 Azar St., Tehran 14145, Iran; 2 Pharmaceutical Department, Food & Drug Laboratory Research Center, Food & Drug Organization (FDO), Ministry of Health, Imam St., Valiasre Cross, Tehran, 1113615911, Iran; 3 Medical Imaging Center, Imam Hospital Complex, School of Medicine, Tehran University of Medical Sciences (TUMS), Keshavaz Blvd., Tehran, 1419733141, Iran; 4 Department of Pharmaceutics, School of Pharmacy, Shahid Beheshti University of Medical Sciences, Valiasre Ave., Niayesh Junction, Tehran, 141556153, Iran

**Keywords:** Nanomagnetic particle, Gadolinium-oxide, Relaxivity, DEG, mPEG-silane

## Abstract

Despite its good resolution, magnetic resonance imaging intrinsically has low sensitivity. Recently, contrast agent nanoparticles have been used as sensitivity and contrast enhancer. The aim of this study was to investigate a new controlled synthesis method for gadolinium oxide-based nanoparticle preparation. For this purpose, diethyleneglycol coating of gadolinium oxide (Gd_2_O_3_-DEG) was performed using new supervised polyol route, and small particulate gadolinium oxide (SPGO) PEGylation was obtained with methoxy-polyethylene-glycol-silane (550 and 2,000 Da) coatings as SPGO-mPEG-silane550 and 2,000, respectively. Physicochemical characterization and magnetic properties of these three contrast agents in comparison with conventional Gd-DTPA were verified by dynamic light scattering transmission electron microscopy, Fourier transform infrared spectroscopy, inductively coupled plasma, X-ray diffraction, vibrating sample magnetometer, and the signal intensity and relaxivity measurements were performed using 1.5-T MRI scanner.

As a result, the nanoparticle sizes of Gd_2_O_3_-DEG, SPGO-mPEG-silane550, and SPGO-mPEG-silane2000 could be reached to 5.9, 51.3, 194.2 nm, respectively. The image signal intensity and longitudinal (*r*_1_) and transverse relaxivity (*r*_2_) measurements in different concentrations (0.3 to approximately 2.5 mM), revealed the *r*_2_/*r*_1_ ratios of 1.13, 0.89, 33.34, and 33.72 for Gd-DTPA, Gd_2_O_3_-DEG, SPGO-mPEG-silane550, and SPGO-mPEG-silane2000, respectively.

The achievement of new synthesis route of Gd_2_O_3_-DEG resulted in lower *r*_2_/*r*_1_ ratio for Gd_2_O_3_-DEG than Gd-DTPA and other previous synthesized methods by this and other groups. The smaller *r*_2_/*r*_1_ ratios of two PEGylated-SPGO contrast agents in our study in comparison with *r*_2_/*r*_1_ ratio of previous PEGylation (*r*_2_/*r*_1_ = 81.9 for mPEG-silane 6,000 MW) showed that these new three introduced contrast agents could potentially be proper contrast enhancers for cellular and molecular MR imaging.

## Background

Magnetic resonance imaging (MRI) is one of the various techniques used widely as imaging tools in clinical diagnosis. Unlike other two methods of computed tomography (CT) and positron emission tomography (PET), MRI has no ionizing radiation, while, with same spatial resolution (SR) as CT, also having a high SR of 0.2 to 0.3 mm compared to 3 mm of PET scan [1,2]. However, the sensitivity and intrinsic contrast of the MRI are low. Imaging contrast depends on signal intensity difference between two adjacent tissues or areas. The effective factors in the signal intensity are proton spin density (*N*), spin–lattice or longitudinal relaxation time (*T*_1_), and spin-spin relaxation or transverse relaxation time (*T*_2_) as shown in Equation 1:

(1) SI∝N.(1−e−TRT1).e−TET2

where TR is the repetition time and TE is the echo time of MRI pulse sequence which determine the contrast between tissues [3].

Contrast agents can modify the signal intensity in different tissues and enhance intrinsic contrast. These are categorized according to the signal intensity produced on *T*_1_- and *T*_2_-weighted images: ‘positive’ (high signal intensity) or ‘negative’ (low signal intensity) [4,5].

Gd^3+^ ions are generally used as a positive contrast agent which has seven unpaired electrons and produce a magnetic moment that is significantly stronger than a proton (nearly 700 times), and its physical properties are suitable for reducing the longitudinal (*T*_1_) and transverse (*T*_2_) proton relaxation times [6]. The efficiency of the contrast agent is determined by relaxivity (*r*_i_) that changes the longitudinal and transverse relaxation times. According to different absorption of agents, this change can result differences among adjacent tissues, as shown in Equation 2:

(2) $${\left(\frac{1}{T_{i}}\right)}_{\text{obs}}={\left(\frac{1}{T_{i}}\right)}_{\text{d}}+r_{i}{[\text{Gd}}^{3+}]\text{i}=1,2$$

where (1/*T*_i_)_obs_ and (1/*T*_i_)_d_ are the relaxation rates (*R*_1_ and *R*_2_, *s*^−1^) of the sample and aqueous solution, respectively; *r*_i_ is longitudinal and transverse relaxivity of the sample (relaxation rates per concentration unit, *s*^−1^ mM^−1^), and [Gd^3+^ is the gadolinium concentration (mM) [7].

Despite the good magnetic properties, the free Gd^3+^ ion is extremely toxic. To reduce its toxicity, it must be complexed by strong organic chelators, e.g., diethylene triamine pentaacetic acid (DTPA) which has been used conventionally in daily MRI examinations [5]. However, these chelates cause low sensitivity and, thus, requiring a high tissue concentration of the contrast agent to be effective for MR imaging.

Recently, studies have shown high efficiency and sensitivity of contrast agents when they have been used in nanoparticles form. The size of the nanoparticles that can be used in MRI is about 3 to 350 nm that might be comparable or smaller than a cell (10 to 100 μm), a virus (20 to 450 nm), a protein (5 to 50 nm), or a gene (2 nm wide and 10 to 100 nm long) [8,9].

For nanoparticles, various coating materials can reduce their toxicity and increase their biocompatibility. As a new surface covering material, this group in a previous study reported some of the primary magnetic properties of diethyleneglycol (DEG) in combination with Gd oxide-based nanoparticles [10]. However, still further researches on the matters of the synthesis procedure, effective size, and agglomeration of gadolinium nanoparticles coated with DEG materials are needed to be done [11-13]. On the other hand, polyethylene glycol (PEG), due to its considerable physicochemical properties, has an especial interest as covering of nanoparticle surfaces [13-17]. Also, it should be noted that PEG has different molecular weights from 350 to 30,000 (and more) Da that could be used alone or in conjunction with other substances such as polylactide-polyethylene glycol and polylactide-co-glycolide [18-20]. For this reason, these two groups of surface conjugate materials (DEG and PEG) could be even useful for covering nanoparticles in biomedical cellular and molecular imaging applications. Therefore, here in continuing our previous works, the assessment of a new supervised DEG synthesis route in addition to a gadolinium PEGylated (PEG) method in comparison to the conventionally Gd-DTPA contrast agent has been determined as the aim of this study. For this purpose, Gd_2_O_3_-DEG was prepared in new synthetic controlled method, and mPEG-silane grafting at the surface of a new core contrast agent (small particulate gadolinium oxide, SPGO < 40 nm) was obtained using two molecular weights of methoxy polyethylene glycol-silane: 550 and 2,000 Da as SPGO-mPEG-silane550 and SPGO-mPEG-silane2000. Physicochemical characterizations and magnetic properties of those three contrast agents in comparison with conventional Gd-DTPA were evaluated to find the optimum method and the more effective contrast agent nanomagnetic for cellular and molecular magnetic resonance imaging.

## Methods

### The synthesis of the Gd_2_O_3_-DEG nanoparticles

In a new supervised polyol route, for the synthesis of gadolinium oxide nanocrystals, 2.5 mmol GdCl_3_-6H_2_O dissolved in 12.5 ml DEG was heated to 140°C until a clear solution was obtained. Then, 3 mmol solid NaOH was dissolved in 6 ml DEG and then added to the Gd-containing solution; the temperature of the mixture was raised to 180°C and held constant for 4 h under reflux and magnetic stirring, yielding a dark yellow colloid. After cooling, the nanocrystals formed were separated and purified from agglomerations or large-size particles by centrifuge filtration for 30 min at 40°C and 2,000 rpm (filters: polyethersulfone, 0.2 μm, Vivascience Sartorius, Hannover, Germany). Free Gd^3+^ ions and excess DEG in the solution were eliminated by 1,000 MW membrane (dialysis tubing, benzoylated, Sigma-Aldrich, USA) for 24 h and by 12,000 MW membrane (dialysis tubing cellulose membrane, Sigma-Aldrich, USA) for 24 h across deionized water, which these sections were not included in our previous study [13].

#### The synthesis of the SPGO-mPEG-silane550 and SPGO-mPEG-silane2000

The process of SPGO-mPEG-silane nanoparticle synthesis was performed using SPGO nanoparticles (<40 nm) purchased from Sigma-Aldrich (99.999% pure). Briefly, a solution was prepared by dissolving SPGO (1 g) and 15 mg ml^−1^ mPEG-silane (mPEG-Silane, MW550, Nanocs, Inc. (MA, USA) or mPEG-Silane, MW 2000, Laysan Bio, Inc. (AL, USA)) in deionized water (10 mL); then, the resulting solution was sonicated at 40°C for 2 h. Large-size particles were separated by centrifugation (2,000 rpm, 30 min) and the suspension was dialyzed (1,000 and 12,000 MW) as described above [21].

### Characterization of the contrast materials

Nanoparticle size measurements were performed three times repeatedly by dynamic light scattering (DLS, Brookhaven Instruments, USA). Also the morphological information of the nanoparticles was done by transmission electron microscopy (TEM, CM120 model, Koninklijke Philips Electronics, Netherlands).

The effects of surface coating in composition with nanomagnetic particles, received from their spectra, was recorded by FTIR spectrometer (Tensor27, Bruker Cor., Germany) over a range between 400 and 4,000 cm^−1^, at room temperature (26°C ± 1°C). Moreover, structural characterization of the SPGO was collected on an X-ray diffractometer (XRD, PW1800, Philips). After filtration and dialysis, the nanoparticle concentrations were determined by induced coupled plasma-atomic emission spectroscopy (Varian-Liberty 150 AX Turbo, USA). In addition, magnetic strength measurements of SPGO and Gd_2_O_3_-DEG were executed with commercial vibrating sample magnetometer (VSM, 7400 model, Lakeshore Cryotronics Inc, OH, USA).

#### Relaxivity measurements

The signal intensity (SI) and longitudinal (*T*_1_) and transverse (*T*_2_) relaxation times were measured by 1.5 T MRI scanner (Siemens AG, Germany) using the head coil. After both types of dialyses (1,000 and 12,000 Da), nanomagnetic concentration measurements done by ICP, then T_1_ and T_2_ changes in aqueous solution for Gd-DTPA, and three synthesized nanomagnetic particles of Gd_2_O_3_-DEG, SPGO-mPEG-silane550 and 2000 were accomplished by diluting them in 5 ml water with Gd concentration at a range of 0.1, 0.3, 0.6, 0.9, 1.2, 1.5, 2, and 2.5 mM (mmol/L). *T*_1_ relaxation time for each sample was obtained by varying repetition times (TR = 100, 200, 400, 600, 2,000 ms) with fixed echo time at TE = 15 ms. Similarly, *T*_2_ relaxation times were measured by varying echo times (TE = 30, 60, 90, 120 ms) and fixed TR = 3,000 ms, and imaging parameters of slice thickness of 5 mm, 1 mm gap, 512 × 384 matrix size, and 25 cm^2^ field of view. Signal intensities were obtained with manually drawn regions of interest for each sample. Relaxation rates of *R*_1_ (1/*T*_1_) and *R*_2_ (1/*T*_2_) were calculated by exponential curve fitting of the signal intensity vs. time (TR or TE) according to Equation 1. After relaxation rate determination for different concentrations, the *R*_1_ or *R*_2_ vs. concentration curve were plotted and, thereby, the relaxivities (*r*_1_ and *r*_2_) as the slope of Equation 2 could be calculated.

## Results

### Characterization of the contrast materials

Table 1 shows the size and polydispersity index (PdI) measurements using DLS; thereby, Gd_2_O_3_-DEG nanoparticles had a hydrodynamic diameter distribution of 5.9 ± 0.13 nm with a PdI of 0.390, while SPGO-mPEG-silane550 and 2000 were 51.3 ± 1.46 nm and 194.2 ± 22.1 nm with PdI of 0.350 and 0.225, respectively. The results showed that when molecular weight increases, the nanoparticle size increase as well. However, despite their different sizes, PdIs of nanoparticles (as an index of the nanoparticle dispersion) had acceptable ranges of less than 0.5.

**Table 1:** **DLS size and PdImeasurements for the threenanoparticle contrast agents**

**Nanoparticle**	**Hydrodynamic diameter**(**nm**)	**PdI**
Gd_2_O_3_-DEG	5.9 ± 0.13	0.387
SPGO-mPEG-silane550	51.3 ± 1.46	0.350
SPGO-mPEG-silane2000	194.2 ± 22.1	0.225

The results show a direct relationship between size and molecular weight.

**Figure 1:**
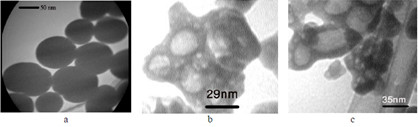
**TEM images of nanoparticles.** TEM images for (**a**) Gd_2_O_3_-DEG, (**b**) SPGO-mPEG-silane550, (**c**) SPGO-mPEG-silane2000. Uniformity and spherical or ellipsoidal shape for Gd_2_O_3_-DEG and agglomeration for two other nanoparticles are observed.

Figure 1 shows the morphology of three wrapped around nanoparticles, while specifically, just images of Gd_2_O_3_-DEG are sharp and uniform such that spherical or ellipsoidal shape of Gd nanomagnetic particles could be visualized separately with clear grains in nano dimensions. The images of two other PEGylated nanoparticles, because of large molecular weights, were agglomerated such that they could not been viewed as sharp as Gd_2_O_3_-DEG nanoparticles among their surface covers.

**Figure 2:**
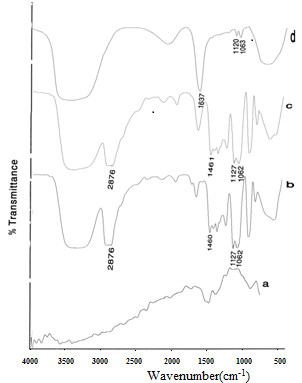
**FTIR spectra for DEGcoatings nanoparticles.** FTIR spectra of (**a**) commercial Gd_2_O_3_ powder (**b**) pure DEG. (**c**) Gd_2_O_3_ nanocrystals prepared by DEG coating without dialysis and centrifuge. (**d**) Gd_2_O_3_ nanocrystals prepared by DEG coating after dialysis and centrifuge. Curves (**c**) and (**d**) show effects of new synthesis route in chemical composition.

FTIR spectra were employed to detect the characteristic bands of different ligands after coating Gd_2_O_3_nanoparticles. Figure 2 shows a comparison of the FTIR spectrum of pure diethyleneglycol with the DEG-coated Gd_2_O_3_ nanocrystals prepared by the polyol method. The bands in DEG at 2,876 and 1,460 cm^−1^ correspond to the symmetric stretching and bending of CH_2_ (Figure 2b). A band at 1,127 cm^−1^ corresponds to C-O stretch, and the broad band of O-H stretch was observed in the 3,100 to 3,500 cm^−1^ range. There are no significant differences between FTIR spectra in Figure 2d, c due to the presence of extra DEG molecules; after the coated Gd_2_O_3_ was cleaned up by dialysis and centrifuge, unreacted DEG has been removed. After coating Gd_2_O_3_ with DEG, shifts in the bands of DEG can be observed in the Gd_2_O_3_-DEG surface. It seems that shifts in the position of CH_2_ and C-O stretching of DEG are due to bonding to Gd_2_O_3_ molecules. Furthermore, the peak shifts from 1,127 to 1,120 cm^−1^ suggest a new configuration for DEG molecules, which oxygen bind to the two Gd atoms and had also been observed by Pedersen et al. [22].

**Figure 3:**
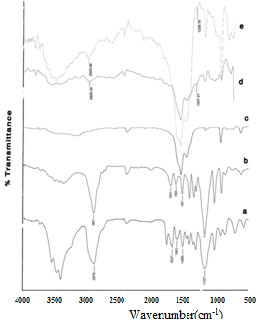
**FTIR spectra for DEGPEGylated nanoparticles.** FTIR spectra of (**a**) mPEG-silane550 powder, (**b**) mPEG-silane2000 powder, (**c**) a commercial SPGO powder, (**d**) mPEG-silane550-coated Gd_2_O_3_ nanoparticles, and (**e**) mPEG-silane2000-coated Gd_2_O_3_ nanoparticles.

FTIR spectrum for mPEG-silane550 is compared with that of the SPGO-mPEG-silane550 in Figure 3a, d, respectively. The FTIR spectrum of the mPEG-silane550 (Figure 3a) displays a peak at 1,284 cm^−1^ corresponding to Si-C stretching vibration. The bands at 2,876 and 1,458 cm^−1^ correspond to the symmetric stretching and bending of CH_2_. The bands at 1,627, 1,107, and 3,100 to 3,500 cm^−1^ correspond to C=O stretching vibration, C-O ether, and N-H stretching vibration, respectively. The band at 1,551 cm^−1^ corresponds to -NH bending vibration in the amide link between the silane and the PEG. The shifts of the characteristic peaks of the mPEG-silane550 to 1,247.21 and 2,925 cm^−1^ (Figure 3d) are strong evidences that PEG bonded to the surface of Gd_2_O_3_ through a reaction of mPEG-silane550 with the nanoparticles surface also been observed by Wu et al. [23]. The bands at 850 and 1,500 cm^−1^ are common between mPEG-silane550 and SPGO-PEG-silane550 after coating SPGO with mPEG-silane550. The spectrum of SPGO-PEG silane2000 is very similar with that of SPGO-PEG silane550, and they have very little differences most likely due to size effects or molecular weight (Figure 3b, e).

**Figure 4:**
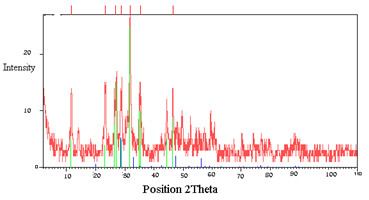
**XRD pattern for SPGO.** Intensities and diffraction angles were according with the standard reference pattern: CSD-Profan database (ref. code: 00-012-0797).

The structural properties of SPGO in Figure 4 showed XRD electron diffraction patterns of nanoparticles that compared with reference code 00-012-0797 of CSD-Profan database in 25°C, included are diffraction angles and intensities that are consistent with standard reference pattern.

**Figure 5:**
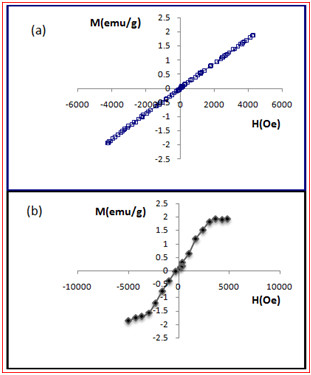
**Magnetometry graphs for SPGOand Gd**_**2**_**O**_**3**_**-DEG.** Graphs of (**a**) SPGO and (**b**) Gd_2_O_3_-DEG magnetization (emu/g) plotted as a function of applied field (Oe). VSM magnetometry shows paramagnetic behavior of SPGO. Also, magnetization curve with S shape (sigmoidal) of Gd_2_O_3_-DEG nanoparticle is shown in (**b**) is similar to superparamagnetic materials.

The magnetic properties of the SPGO and Gd_2_O_3_-DEG nanoparticles were measured by VSM at room temperature. The relative magnetization curves vs. applied field were plotted in Figure 5. For paramagnetic, diamagnetic, and superparamagnetic materials, when the applied magnetic field is removed, they should exhibit no coercivity and remanence. Also, paramagnetic materials have a linear relationship between magnetization (*M*) and applied field (*H*) with positive slope. As shown in Figure 5a, SPGO particles revealed paramagnetic properties. Also, magnetization curve with S shape (sigmoidal) of Gd_2_O_3_-DEG nanoparticle is shown in Figure 5b, which is similar to superparamagnetic materials. Thereby, the difference between SPGO and Gd_2_O_3_-DEG in relation to covering Gd_2_O_3_ with DEG could be seen clearly in Figure 5b.

### Relaxivity measurements

**Figure 6:**
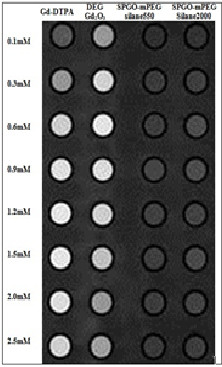
**Arrangement of nanoparticle tubesfor imaging and relaxometry.** Signal intensities for Gd_2_O_3_-DEG were more than Gd-DTPA and two other nanoparticles. The quantitative variation results of signal intensities in Figure 8b are in complete accordance with the image visualization in relation to *in vitro* dilutions of the three materials. Signal intensities displayed a relatively steep increase when approaching a more gradual increase, thereafter.

**Figure 7:**
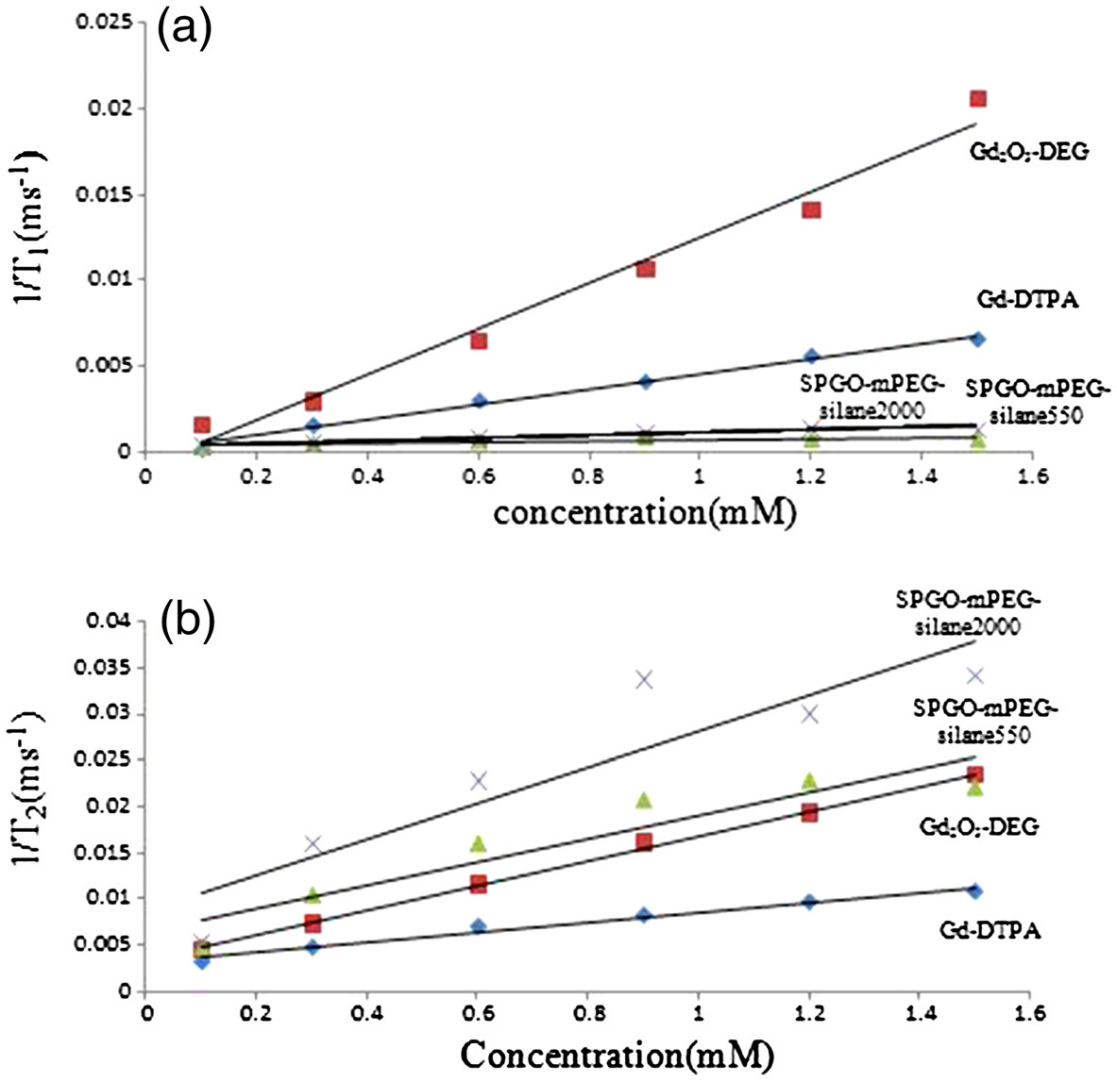
**Longitudinal(*R***_**1**_**)(a)and transversal(*R***_**2**_**)(b)relaxation rates vs**. **concentration.** The slope of the curve or relaxivity (*r*_1_ and *r*_2_) was obtained for Gd-DTPA (diamond), Gd_2_O_3_-DEG (square), SPGO-mPEG-silane550 (triangle), and SPGO-mPEG-silane2000 (cross). The solid lines represent the linear regression of the data. Gd_2_O_3_-DEG had longitudinal proton relaxivity at least 2.5 times higher than Gd-DTPA, whereas *r*_1_ for SPGO-mPEG-silane550 and 2000 was less compared with Gd-DTPA.

Nanoparticle tubes were prepared by certain concentrations (Figure 6). *R*_i_ (1/*T*i, *i* = 1, 2) vs. Gd concentration curve were plotted, and the slope of the curve or relaxivity (*r*_i_) was obtained for each nanoparticle (Table 2). Gd concentration shows a linear relationship up to 1.5 mM with a good of fit *r* > 0.98 according to Equation2 (Figure 7).

**Table 2:** **Results of relaxometry forthree nanoparticle contrast agentsand Gd**-**DTPA**

**Nanoparticle**	***r***_**2**_**/*r***_**1**_	***r***_**2**_**(mM**^**−1**^**s**^**−1**^**)**	***r***_**1**_**(mM**^**−1**^**s**^**−1**^**)**
Gd-DTPA	1.13	5.14	4.55
Gd_2_O_3_-DEG	0.89	11.81	13.31
SPGO-mPEG-silane550	33.34	26.34	0.79
SPGO-mPEG-silane2000	33.72	33.72	1.00

Longitudinal relaxivity (*r*_1_) of Gd-DTPA PEGylated nanoparticles (SPGO-mPEG-silane550 and 2000) was smaller than that of Gd_2_O_3_-DEG.

**Figure 8:**
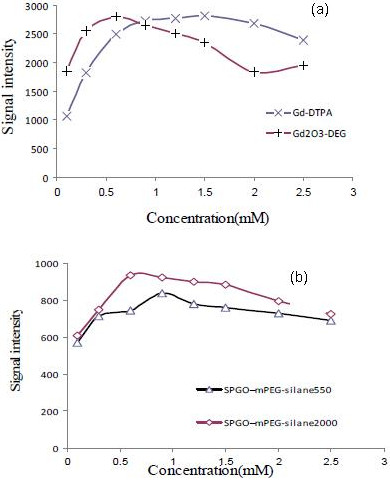
**Signal intensities for contrastagents.** Relative signal intensities in (**a**) Gd-DTPA and Gd_2_O_3_-DEG. (**b**) SPGO-mPEG-silane550 and SPGO-mPEG-silane2000 (TR = 600 ms and TE = 15 ms). Maximum signal intensity for Gd_2_O_3_-DEG was obtained (0.6 mM), whereas it was 1.5 mM for Gd-DTPA.

Figure 7a shows the longitudinal relaxation rates (1/*T*_1_) for the used materials. Gd_2_O_3_-DEG had longitudinal proton relaxivity at least 2.5 times higher than Gd-DTPA, whereas *r*_1_ for SPGO-mPEG-silane550and 2000 was less compared with Gd-DTPA. That is why, unlike Gd_2_O_3_-DEG and Gd–DTPA, *R*_1_ relaxation rates of SPGO-mPEG-silane550 and 2000 did not change considerably with concentration. In Figure 8b, for all of nanoparticle materials and Gd-DTPA, the change of Gd concentration led to the increase of transverse relaxation rates (1/*T*_2_), while this effect is significantly higher for SPGO-mPEG-silane550 and 2000 compared to Gd_2_O_3_-DEG and Gd–DTPA (Figure 7b, Table 2).

#### Determination of maximum signal intensities for different concentrations

Figure 8 shows the signal intensity curves for Gd-DTPA, Gd_2_O_3_-DEG, SPGO-mPEG-silane550, and SPGO-mPEG-silane2000 using TR/TE = 600/15 ms. The maximum signal intensities for Gd_2_O_3_-DEG, SPGO-mPEG-silane550, and SPGO–mPEG-silane2000 were found in 0.6, 0.6, and 0.9-mM concentrations, respectively.

## Discussion

Contrast agents can modify the signal intensity in different tissues to enhance their contrast and improve the low sensitivity of magnetic resonance imaging. The efficiency of the contrast agents according to different absorption of agents is determined by *r*_i_ that changes the longitudinal and transverse relaxation times to result differences among adjacent tissues. These changes are categorized according to the signal intensity produced on *T*_1_ and *T*_2_-weighted images: ‘positive’ known as high signal intensity or ‘negative’ as low signal intensity. Recently, studies have shown high efficiency and sensitivity of contrast agents when they have been used in nanoparticle forms. To have higher relaxivity, reduce toxicity, increase biocompatibility and half-life, besides preventing the nanoparticle aggregations, contrast agents in MRI should be coated with various materials. Different factors could affect the sizes of nanoparticles including type of the core, coating molecular weights, nanoparticle aggregation and, thereby, the synthesis route. Theoretically, by increasing molecular weights of nanoparticle coatings, their average size could be increased as well [24]. For this reason, in this study, we investigated magnetic properties of three Gd-based nanoparticles with different coatings of DEG, mPEG-silane550, and mPEG-silane2000 comparing to conventionally extracellular Gd-DTPA contrast agent. For nanoparticle synthesis, two different methods were used. Firstly, the preparation and coating of Gd_2_O_3_ by previous polyol route besides 0.2-μm filtration, and two 1,000 and 12,000 Da dialysis membranes led to reach the good and desirable smaller size of approximately 5 nm of gadolinium crystal nanoparticles covered by DEG in Gd_2_O_3_-DEG compounds. Secondly, for mPEG-silane550 and mPEG-silane2000, despite using filtration and sonication after PEG coating method for elimination aggregated particles prior to DLS measurement, PEGylated nanoparticles even still had relatively larger sizes of approximately 51.3 and approximately 194.2 nm. For this, part of that increase size should be due to the effect of their molecular weights. In our study, molecular weights of three materials were as follows: MWSPGO-mPEG-silane2000 > MWSPGO-mPEG-silane550 > MW Gd_2_O_3_-DEG. As seen in Table 1, the measured particle sizes have an incremental behavior as the molecular weight has increased, which are in accordance with their appearance in related TEM images.

Magnetic properties in MRI were related to relaxivities (*r*), especially, *r*_2_/*r*_1_ ratio that defines the potential for being a positive or negative contrast agent. Meanwhile, several studies have investigated the size effects on magnetic properties and relaxivities, e.g., SPIO nanoparticles with hydrodynamic diameters of 9, 12, and 15 nm had *r*_2_/*r*_1_ ratio of 2.75, 5.95, and 13.08, respectively [22,23]. Some other studies have also showed that the *r*_2_/*r*_1_ ratio increases with larger sizes of nanoparticles [14,25,26]. Consequently, in this study, the changes of coating materials with various molecular weights on a similar core were also studied in terms of *r*_2_/*r*_1_ ratios which have been shown in Table 2. Thereby, it is clear that those *r*_2_/*r*_1_ ratios for Gd_2_O_3_-DEG were much lower than that of other two PEGylated materials. Meanwhile, even for SPGO-mPEG-silane2000, the said ratio was a bit higher than SPGO-mPEG-silane550.

For positive contrast agents, *r*_2_/*r*_1_ ratio is described to be 1 to 2 and for negative ones; however, it is between 2 and 40 [21]. Thus, in our study, Gd_2_O_3_-DEG (with *r*_2_/*r*_1_ ratio = 0.89) could reveal good results as a positive contrast agents even better than Gd-DTPA (with *r*_2_/*r*_1_ ratio = 1.13) [10-12], that is in part because of such small size nanoparticles that could be yielded in the new synthetic method in this research. However, *r*_2_/*r*_1_ ratios for PEGylated nanoparticles are relatively high. In one study, PEGylated SPGO with higher MW (MW = 6,000 Da) resulted to an *r*_2_/*r*_1_ ratio equal to 81.6 [20]. In this study, we used polymers with a lower molecular weight (i.e., 550 and 2,000) and so the *r*_2_/*r*_1_ ratios could be reached to 33.34 and 33.72, respectively. These decreased ratios in our study should be mostly related to the selected lower molecular weight materials. Furthermore, the relaxivity results in Figure 7a, b indicate that Gd_2_O_3_-DEG nanoparticles (with lower *r*_2_/*r*_1_ ratio) as positive contrast agents are clearly more appropriate than Gd-DTPA. SPGO-mPEG-silane550 and 2000 due to having both high *r*_2_ and high *r*_2_/*r*_1_ ratio appear to be proper contrast agents for *T*_2_-weighted MR imaging methods, as well.

According to Equations 1 and 2, signal intensities change with *T*_1_, *T*_2_, and the concentration of contrast agents. Therefore, short *T*_1_ leads to a signal increase, whereas, short *T*_2_ decreases the signal. A maximum signal occurs at intermediate concentrations; such expectations could be seen clearly in Figure 8a, b. In addition, the maximum signal intensity for Gd_2_O_3_-DEG occurred at similar daily clinical concentration relative to Gd-DTPA with similar intensity (0.6 mM near to 0.1 mM; Figure 8a). Also, signal intensities for SPGO-mPEG-silane550 and SPGO-mPEG-silane2000 were much less than the two other contrast agents (Figure 8b). This is another conformation that they can be considered as negative or *T*_2_-wieghted contrast agents. This could be remained for future experiment for them to be compared with other negative contrast agents such as iron oxide-based ones.

## Conclusions

The synthesis controlled method making use of dialysis, filtration, and sonication could have direct effect on the nanosize scale and magnetic characterization of nanoparticles, consequently on their *r*_2_/*r*_1_ ratio as providing and giving them a positive or negative signal properties of contrast agents. Thereby, in our study, the Gd_2_O_3_-DEG with *r*_2_/*r*_1_ lower than Gd-DTPA and other previously synthesized Gd_2_O_3_-DEG could be achieved. Moreover, for preparation of PEGylated contrast agents, polymers with lower molecular weights could potentially have better contrast properties as behaving like negative contrast agents that should be compared with other similar negative ones.

Therefore, among different group coating materials, DEG and PEG, due to their considerable properties and not having fixed sizes (different molecular weights), were selected as useful surface covering of nanomagnetic particles that could reveal noticeable relaxivity, magnetic property, and signal intensity that are proper for cellular and molecular MRI applications that would be remained for future *in vivo* studies.

## Competing interests

The authors declare that they have no competing interests.

## Authors' contributions

GA and NRA designed the study, carried out all of the experimental work and data acquisitions, and drafted the manuscript. SH and EG performed the synthesis and the interpretation of nanoparticles' chemical structure. HRM contributed in drug regulations consultancy. RZ participated in material characterizations. BR carried out the magnetic resonance imaging protocols. All authors read and approved the final manuscript.

## Authors' information

NRA was born in 1960 in Shiraz, Iran, received his BSc in Nuclear Physics from Shiraz University, Shiraz, Iran, in 1986, his MSc and Ph.D. in Medical Physics (Medical Imaging) from Nagoya University, Nagoya, Japan in 1995. At present, he is a professor in the Medical Physics at the Department of Medical Physics & Biomedical Engineering, School of Medicine, Tehran University of Medical Sciences (TUMS). His research interests during Ph.D. graduation was in computer-aided diagnostic (CAD) systems for the detection of cancer on digital mammograms, leading to development of software algorithms and CAD systems for mammographic cancer detection. As a PIP member of the American Association in Medical Physics (AAPM) and other scientific forums, he published many articles and several books in Medical Imaging aspects, achieved awards in innovation, scientific hypothesis and inventions, and paper awards in world conferences. Recently, his research interests have been focusing on the development of nanomagnetic particles for MRI applications. The development of the present work in combination with the magnetoliposomes as tumor cell tracking and drug delivery system for liver specific target detection has been accepted and will be presented in RSNA2012.

SH was born in 1960 in Abadan, Iran, and received her BSc and MSc in Analytical Chemistry from Shiraz University, Shiraz, Iran, in 1990, and her Ph.D. in Pharmaceutics from Nagoya University in Japan in 1995. Her Ph.D. research was related to pharmacokinetics of drugs, especially drug characterization in brain distribution. At present, she is an associate professor in the Pharmaceutical Department, Food & Drug Laboratory Research Center, Food & Drug Organization (FDO), Ministry of Health, Tehran, Iran. She is the head of QC approving of herbal medicine and supplements in Iran FDO and also has been the lecturer of GLP and GMP at the universities while performing many workshops for drug factories. Her recent research interest is in the synthesis and analytical method development of nanosized molecules for drug delivery applications.

HRM received his Pharm D from Tabriz University and his Ph.D. in Pharmaceutics from the University of Bradford, England in 1995. He has been a member of the Fellowship in Liposomal Gene Delivery in Cancer, University of Alberta, Canada. At present, he is a professor of Pharmaceutics in School of Pharmacy, Shahid Beheshti University of Medical Sciences, Tehran, Iran. His research interests have been related to transdermal drug delivery, modeling biological barriers, gene delivery, liposomal drug delivery, and lyotropic liquid crystals. He has been also the Director General of some drug factories.

GA is a Ph.D. candidate who received his BSc in Physics from Bu Ali Sina University in 1991, his MSc in Medical Physics from Tehran University of Medical Sciences (TUMS) and now is going to graduate in Medical Physics from Tehran University. He has been the employee and the lecturer of Medical & Radiation Physics in School of Medicine of Hamadan University of Medical Sciences since 2000. His research interests during his MSc has been related to medical imaging and now during his PhD graduation he has been involving with the development of magnetic nanoparticle sizes for MRI applications.

RZ, a Ph.D. candidate, received his BSc in Physics from Tehran University of Medical Sciences in1999, his MSc in Medical Physics from Tabriz University of Medical Sciences and now is going to graduate in Medical Physics from Tehran University. His research interest is related to magnetoliposome nanosize particles application for MRI applications.

EG received her BSc in Chemistry from the Islamic Azad University of Tehran University and her MSc from the University of Zanjan (ZNU). She is a research fellow in Food & Drug Laboratory Research Center, Food & Drug Organization (FDO), Ministry of Health, Tehran, Iran. Her research interests in MSc has been in the preparation of gold nanoparticles coated by amino acids and the study of the kinetically influence tubulin protein *in vitro*, and at present, is working on the synthesis development for contrast agent nanosize particles for MRI applications.

BR received his BSc in Radiology from Iran Medical University in 1990 and his MSc in Medical Physics from Iran Medical University in 2001. At present, he is an employee of Medical Imaging Center in Emam Hospital Complex, School of Medicine. He has been contributing to so many researches with different departments and researchers; he is qualified in developing different MRI imaging protocols. At present he is responsible for performing new and proper MRI data acquisitions in nanomagnetic contrast agents and MRI neuroimaging applications.
